# Time-Dependent Effect of Chitosan Nanoparticles as Final Irrigation on the Apical Sealing Ability and Push-Out Bond Strength of Root Canal Obturation

**DOI:** 10.1155/2020/8887593

**Published:** 2020-07-15

**Authors:** Diatri Nari Ratih, Nikita Ika Sari, Pribadi Santosa, Nofa Mardia Ningsih Kaswati

**Affiliations:** ^1^Department of Conservative Dentistry, Faculty of Dentistry, Universitas Gadjah Mada, Yogyakarta 55281, Indonesia; ^2^Specialist Study Program of Conservative Dentistry, Faculty of Dentistry, Universitas Gadjah Mada, Yogyakarta 55281, Indonesia; ^3^Nanotech Indonesia, Serpong, Tangerang Selatan, Banten 15314, Indonesia

## Abstract

**Materials and Methods:**

Fifty-six premolars were used in this study and divided by two evaluations: 28 teeth for apical sealing ability and 28 others for bond strength. Each study was assigned randomly into two groups of fourteen teeth: Group-1, final irrigation with 17% EDTA; Group-2, with 0.5% chitosan nanoparticles. Each group was further divided into two groups of 7 each: Group-A, final irrigation was applied for 1 minute; Group-B, for 3 minutes. All teeth were obturated with epoxy resin-based sealer and gutta-percha. In the apical sealing ability study, the obturated teeth were immersed in 2% methylene blue and observed under a stereomicroscope (8x magnification). In the bond strength study, the teeth were tested using the push-out technique and observed under a stereomicroscope (40x magnification) to determine the failure type. Data from each evaluation were analysed with two-way ANOVA followed by the LSD test.

**Results:**

Final irrigation using 0.5% chitosan nanoparticles produced the same apical sealing ability and bond strength as 17% EDTA (*p* > 0.05). A significant difference occurred between application times (*p* < 0.05). The failure type was observed predominantly as cohesive, and the least was adhesive.

**Conclusion:**

Regardless of the final irrigation solution used, 3-minute application time produced greater apical sealing ability and push-out bond strength than 1-minute application time.

## 1. Introduction

The success of root canal treatment depends on endodontic triads, which include biomechanical preparations (cleaning and shaping), chemical root canal cleaning using irrigation solutions, intracanal medicaments, and obturation of the root canal. Among those triads, cleaning using irrigation solutions is a crucial phase during root canal treatment. The goal of root canal irrigation is to aid in cleaning of root canals from vital or necrotic pulp tissue remnants as well as to eliminate microorganisms and their products [1, 2].

The endodontic instruments used manually or mechanically will create smear layers on the root canal wall and smear plugs in the dentinal tubules. The thickness of the smear layer is formed in the ranges from 0.5 to 2 *μ*m. Although the thickness is only a few microns, the presence of smear layers may impede sealer infiltration into dentinal tubules and sealer adhesion on the wall of the root canal dentin [3].

The obturation of root canal systems was intended to achieve an adequate sealing ability and adhesion to dentin. An ideal sealer for root canal obturation should attach tightly to both dentin and gutta-percha [4]. The bond strength of the endodontic sealer to dentin is important to preserve the sealing ability of the root canal since there is a direct association between the sealer bond and leakage [5].

Many types of sealers for root canal obturation are available in the market, and resin-based sealers are very popular recently [6]. This sealer can adhere adequately to the root canal dentin, resulting in good adaptation of sealers to the root canal dentin. The adaptation of the resin sealer is influenced by smear layers, and according to previous studies, the smear layer can decrease resin sealer adaptation to the root canal walls [7]. Therefore, cleaning of the smear layers from the root canals is advocated to improve sealer adhesion to the root canal dentin, resulting in both good apical sealing ability and bond strength, as a consequent optimally successful root canal treatment accomplished [8].

Irrigation solution commonly used in the clinic is 2.5–5% of sodium hypochlorite (NaOCl). NaOCl has the capability to eliminate organic tissues but is unable to remove inorganic tissues from the smear layers on the root canal walls; therefore, other irrigation solutions require to be utilized, which is called a final irrigation solution with the purpose to eliminate inorganic tissues of smear layers [9].

Ethylenediaminetetraacetic acid (EDTA) is often used in the clinic and as the gold standard for final irrigation solution since its ability to chelate with calcium ions in dentin, thus dissolving the inorganic component from smear layers [10]. However, the prolonged application time of EDTA to the dentinal wall can cause dentin erosion and reduce microhardness [11]. Besides, EDTA has no antibacterial properties and a pollutant [12].

Recently, chitosan has been widely used in dentistry because of many beneficial properties, such as biocompatibility, biodegradability, bioadhesion, and nontoxic. Chitosan is a polysaccharide, and it is attained from chitin, which is obtained from the seashell of crustaceans and shrimps, through the process called deacetylation [13, 14]. In addition, in an acid environment, chitosan has the capability to chelate with various metal ions [15].

Previous studies have suggested that chitosan has high antibacterial properties against *Enterococcus faecalis* and *Candida albicans* [16]. A previous study by Carpio-Perochena et al. [17] reported that chitosan has the chelation ability; hence, it is able to dissolve the smear layer. Chitosan, in the form of nanoparticles, has been undertaken to optimize the effectiveness of chitosan as root canal irrigation because it has better absorption and penetration into dentinal tubules [18].

The controversy of the final irrigation solution application time to the dentinal wall still exists in removing the smear layer. Several authors suggested preserving the smear layer since it can prevent apical microleakage [19]. However, other authors recommended the smear layer removal as it consists of microorganisms that can cultivate in the dentinal tubules [20]. Therefore, the application time of the final irrigation solution is the essential factor in removing the smear layer to enhance the adhesion of root canal obturation; as a result, the success of root canal treatment can be achieved [21].

Because chitosan has many advantages, while EDTA has a limitation as a final irrigation solution, therefore, the former has potential to be a final irrigation solution in the future, and it requires to be investigated further [14, 17]. Many factors influence the efficacy of final irrigations. One of the factors is the application time of final irrigation solution. In addition, to date, no agreement occurred in the apt application time of final irrigation solution on the root canal dentin, which may enhance the apical sealing ability and bond strength of root canal obturation. Conversely, only a few studies are available in the literature that has investigated the application time of chitosan nanoparticles as final irrigation on apical sealing ability and push-out bond strength. Thus, this present study aimed to evaluate the time-dependent effect of 0.5% chitosan nanoparticles as the final irrigation solution on the apical sealing ability and bond strength of root canal obturation.

## 2. Materials and Methods

In the present study, two evaluations were undertaken: apical sealing ability and microhardness of root canal dentin. This study protocol was approved by the Institutional Ethics Committee, no. 00182/KKEP/FKG-UGM/EC/2019. This study used fifty-six intact, single, straight roots, and caries-free premolar teeth, which had an initial file # 20 (Dentsply Maillefer, Ballaigues, Switzerland). Twenty-eight teeth were used for apical sealing ability evaluation, and 28 others were used for bond strength evaluation. The crowns of the teeth were removed, leaving 14 mm root length. Working length was determined by subtracting 1 mm of root length. The root canals were prepared with rotary files (ProTaper Universal, Dentsply Maillefer) up to F3 using the crown-down technique according to the manufacture's protocol. Throughout shaping and cleaning, the application of 2 mL 2.5% NaOCl (Golden Falcon, Dubai, UAE) as an irrigant solution was performed at each change of instrument. Afterwards, all teeth were rinsed using 5 mL of distilled water.

All teeth for each evaluation were then randomly assigned into two groups of fourteen specimens. Group-1 was final irrigated with 17% EDTA (Pulpdent, Watertown, MA, USA). Group-2 was final irrigated with the 0.5% chitosan nanoparticle. Each group was further divided into two groups of 7 specimens: Group-A, application time for 1 minute of final irrigant solution, and Group-B, application time for 3 minutes. Each volume of irrigant solution used was 50 *µ*L and was placed into the canal with a 30-gauge needle, which placed approximately 2 mm into the root canal. Finally, root canals were flushed using distilled water (5 mL) and were dried using paper point # 30.

All root canals were obturated with gutta-percha #F3 (ProTaper, Dentsply Maillefer) and epoxy resin-based sealer (AH Plus, Dentsply, DeTrey, Konstanz, Germany). Radiographic images were undertaken to observe the hermetic obturation. Specimens were kept in an incubator with a temperature of 37°C and 100% humidity for 7 days to allow the setting of the sealer.

The 0.5% chitosan nanoparticle solution was created by dissolving 0.5 grams of chitosan powder (PT NHI, Tangerang, Indonesia) in 1% acetic acid with a volume of 100 mL. Chitosan was synthesised from shrimp shells (degree of deacetylation >75%) using the ionic glass method and polyanion tripolyphosphate (TPP) as a crosslinker. The mixture was stirred with a magnetic agitator for 2 hours to obtain a homogenous solution [22].

### 2.1. Apical Sealing Ability Evaluation

**Figure 1:**
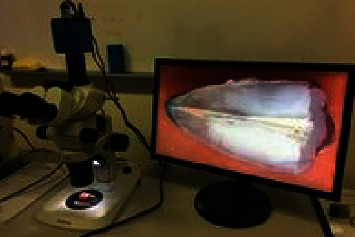
Methylene blue penetration was observed under a stereomicroscope with 8x magnification. The penetration of the methylene blue solution was observed in the apical region.

After root canal obturation, twenty-eight specimens for apical sealing ability evaluation were covered with nail polish on the root surface (Revlon, New York, NY) except for 1 mm of the root apex and immersed in 2% methylene blue for 7 days. Following immersion, specimens were cleaned from nail polish and then were split perpendicularly to the tooth axis in the buccolingual direction to produce the mesial and distal sections (Buehler Ltd., Evanston, IL, USA). One of the regions which had the longest methylene blue penetration was observed under a stereomicroscope (Olympus SZX7, Olympus Corp., Tokyo, Japan) with 8x magnification (Figure 1). The microscope was connected to the computer monitor. Apical sealing ability was measured by observing the penetration of methylene blue solution from apical to the coronal direction and measured by the Image tool program (UTHSCSA ImageTool Version 3.0). Measurements of methylene blue penetration were made three times of each specimen. The results of all three measurements were averaged, and the shortest methylene penetration indicated the best apical sealing ability.

Data obtained were analysed using a two-way analysis of variance (ANOVA) followed by the LSD post hoc test at the 95% level of confidence.

### 2.2. Bond Strength Evaluation

Following root canal obturation, the apical third of other twenty-eight specimens for push-out bond strength evaluation was sectioned horizontally into slices with a thickness of 2 mm using a diamond saw microtome (SP 1600 microtome, Leica, Nubloch, Germany). The lumen of the root dentin was instrumented with Gates-Glidden burs (Dentsply, Maillefer), sizes 2 to 5, to attain a uniform diameter of 1.3 mm.

The push-out test was conducted using a 1 mm diameter stainless plugger (Dentsply, Maillefer), attaching to a universal testing machine (Pearson Parke Equipment Ltd., London, UK). Each specimen was positioned on a metal slab with a central hole to permit the free movement of the plugger. A load was applied by employing a descending pressure on the surface of obturation material using a 1 mm diameter cylindrical stainless steel plugger at a crosshead speed of 1 mm/minute [23]. To ensure that the plugger only contacts to the obturation material, hence the plugger had a clearance of approximately 0.2 mm from the margin of the dentinal wall. The maximum load applied to obturation material at the time of displaced was recorded in Newton (N).

To express the bond strength in megapascals (MPa), the recorded value was divided by the adhesion surface area of the obturation material calculated by 2*π*r × h, where *r* is the root canal radius and *h* is the thickness of the root dentin slice (in mm). After the push-out test, each specimen was observed under a stereomicroscope (Olympus SZX7, Olympus Corp., Tokyo, Japan) at 8x magnification to evaluate the bond failure type. Each specimen was characterized into one of the three failure modes: adhesive failure at the obturation material/dentin interface, cohesive failure within obturation material, or mixed failure [4]. Data obtained were analysed using a two-way ANOVA followed by the LSD post hoc test at the 95% level of confidence.

## 3. Results

Tables 1 and 2 show that the final irrigation using 0.5% chitosan nanoparticles had the higher either apical sealing ability or bond strength, followed by 17% EDTA. However, both irrigation solutions were not a statistically significant difference (*p* > 0.05). Regarding application time, it was demonstrated that the application of 3 minutes produced the greater apical sealing ability and bond strength than 1 minute (*p* < 0.05) regardless of the final irrigation solution used.

**Figure 2:**
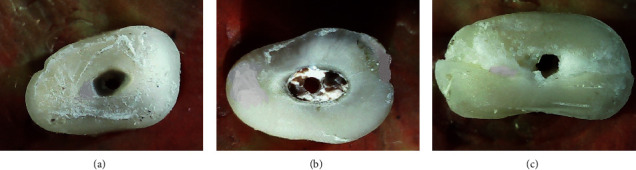
The stereomicroscope examination with a magnification of 8x of representative specimens revealed adhesive failure, which showed that the root canal wall had no attached sealer (a); cohesive failure, which exhibited that almost the entire root canal wall was still covered with sealer (b); and mixed failure (mixed between adhesive and cohesive failure), which revealed that some parts of the root canal wall were covered with sealer (c).

The stereomicroscope observations of specimens (Figure 2) after the push-out test revealed that the highest failure was cohesive (60.71%) and the lowest was an adhesive failure (17.86%) (Table 3).

## 4. Discussion

This study demonstrated that final irrigation with both 17% EDTA and 0.2% chitosan nanoparticles with the application time of 3 minutes produced greater apical sealing ability and bond strength than 1-minute application time. It seemed that EDTA and chitosan nanoparticles act as a chelation agent to the smear layer, primarily to inorganic tissue, which always forms after root canal preparation with files [19]. The results of this present study were in agreement with a previous study, which reported that chitosan removes the smear layer as effective as EDTA [24, 25]. Asharf et al. [26] reported that to obtain a good apical sealing ability required smear layer elimination from the internal surface of root canal dentin. However, many factors might affect apical sealing ability, such as obturation techniques and sealer materials [2]. The smear layer formed must be removed for any of the following reasons: it can block the irrigation material and prevent sealer infiltration into either the dentinal tubules or fibrillar spaces of intertubular dentin of the root canal [27]. The smear layer also consists of bacteria and its products as well as the necrotic tissues, which may cause bacteria to enter deeper into the dentinal tubules, and it can prevent adaptation between obturation material and root canal walls, resulting in poor apical sealing ability and bond strength [28, 29].

This study used adhesive material of epoxy resin as a sealer. Bonding of this type of the sealer to the root canal wall is predominantly influenced by the existence of smear layers because smear layers are able to disturb the adhesion of adhesive materials to root canal walls [4]. Final irrigation using 17% EDTA and 0.5% chitosan nanoparticles seems to dissolve the smear layer, especially the inorganic substance, although in the differential mechanism [14, 30]. EDTA has the ability to create chelation with calcium ions in dentin and causing dentin to dissolve. It can decalcify dentin with a depth of 20–50 *µ*m ranging between two and three minutes [12].

Chitosan also has chelation properties as EDTA. Though it is not fully considered, the chelating mechanism to dentin, the adsorption, ion exchange, and chelation may be assumed to manipulate the connection between the metal ions and the chelating agents [31]. Moreover, this connection is stipulated by the pH of the solution, ion involvement, and chitosan chemical structure [32].

Chitosan nanoparticle is hydrophilic; hence, it can maintain tight contact and can be adsorbed to the root canal dentin. Chitosan has an enormous amount of hydroxyl and amino groups, which create chitosan becoming cationic, inducing ionic interactions with calcium dentin ions. The amino group in chitosan can be protonated, which leads to the withdrawal of other molecules for adsorption into the root canal dentin, resulting in entering more in depth into the dentinal tubules [14]. On the contrary, the epoxy resin is hydrophobic; hence, the hydrophilic nature of chitosan is able to increase the wetting ability of the sealer material to the root canal wall, which has an irregular surface due to the opening of the dentinal tubules after instrumentation and irrigation [29, 33]. Furthermore, nanosized chitosan particle size can amplify the flow of irrigation solution into the dentinal tubules inducing smear layer removal increase; as a result, adequate bond strength can be achieved between the obturation material and root canal wall [4, 8].

This study also demonstrated that the sealing ability and bond strength of root canals, which were irrigated using 17% EDTA, were not significantly different from 0.5% chitosan nanoparticles (*p* > 0.05). However, the application time of 3 minutes produced greater sealing ability and bond strength than 1 minute regardless of the final irrigation solution used. This phenomenon probably due to the two irrigation materials have the same chelation ability in eradicating the smear layer [8]. The cleanliness of the root canal wall from the smear layer increases bond strength to dentin and reduces microleakage for sealers by enhancing the sealer penetration into the dentinal tubules resulting in mechanical interlocking to the canal walls [8]. Several previous studies were in accordance with this study which stated that 0.2% chitosan produced the same bond strength as 17% EDTA [34, 35] and greater sealer penetration with a variety of application times [36, 37].The advantage of chitosan compared to EDTA as the final irrigation material is chitosan has antibacterial properties, while EDTA has no antibacterial properties [16], although antibacterial properties of chitosan were not tested in this study.

The result of the present study showed that the application time is a factor that affects the removal of the smear layer. The longer the application time generated, the greater both the apical sealing ability and bond strength. This phenomenon might be associated with fewer patent tubules in the apical region compared to the other areas of the root canal [4]. In addition, the smear layer is more challenging to be eliminated in the apical part of the root canal since the diameter of the root canal is getting narrower in the apical third [23]. Therefore, the longer application time of irrigation solution is more effective in removing the smear layer in the apical third of root canal, which in turns, greater sealing ability and bond strength accomplished [33].

This study used chitosan in the form of nanoparticles due to its deeper absorption and penetration into dentinal tubules and intertubular dentin; as a consequence, the removal of the smear layer increases [27]. The chitosan percentage of 0.5% was used in this study since, according to previous investigators, 0.5% chitosan nanoparticles were effective in removing smear layers in coronal, mid, and third apical root canals [4].

## 5. Conclusions

Within the limitations of this study, it was concluded that regardless of the final irrigation solution used, 3-minute application time produced greater apical sealing ability and pushout bond strength than 1-minute application time. Thus, the application time of 3 minutes is suggested to be undertaken when using the 0.5% chitosan nanoparticle for the final irrigation solution.

## Figures and Tables

**Table 1:** Mean and standard deviation of the sealing ability of root canal obturation (in mm).

Final irrigation	Application time	Mean ± standard deviation
17% EDTA	1	5.17 ± 1.35^a^
	3	2.06 ± 0.43^b^

0.5% chitosan nanoparticle	1	4.88 ± 1.62^a^
	3	1.97 ± 0.61^b^

^*∗*^Different letters indicate that there were statistically significant differences.

**Table 2:** Mean and standard deviation of the pushout bond strength of the root canal obturation (in MPa).

Final irrigation	Application time	Mean ± standard deviation
17% EDTA	1	6.24 ± 1.66^a^
	3	9.37 ± 1.06^b^

0.5% chitosan nanoparticle	1	7.89 ± 2.02^a^
	3	10.19 ± 1.57^b^

^*∗*^Different letters indicate that there were statistically significant differences.

**Table 3:** Percentage of failure modes following the pushout bond strength test between obturation material and root canal dentin.

Final irrigation	Application time (min)	Adhesive (%)	Cohesive (%)	Mixed (%)
17% EDTA	1	42.86	42.86	14.28
	3	28.57	71.43	0

0.5% chitosan nanoparticle	1	14.28	42.86	42.86
	3	0	85.72	14.28

Total		17.86	60.71	21.43

## Data Availability

The data used to support the findings of this study are available from the corresponding author upon request.
